# Distinct evolutionary patterns of endemic and emerging parvoviruses and the origin of a new pandemic virus

**DOI:** 10.1073/pnas.2515274123

**Published:** 2026-04-14

**Authors:** Robert A. López-Astacio, Brian R. Wasik, Hyunwook Lee, Ian E. H. Voorhees, Wendy S. Weichert, Oluwafemi F. Adu, Laura B. Goodman, Susan L. Hafenstein, Uwe Truyen, Colin R. Parrish

**Affiliations:** ^a^Baker Institute for Animal Health, Department of Microbiology and Immunology, College of Veterinary Medicine, Cornell University, Ithaca, NY 14853; ^b^The Hormel Institute, Department of Biochemistry, Molecular Biology and Biophysics, University of Minnesota, Medical Research Center, Austin, MN 55912; ^c^Baker Institute for Animal Health, Department of Public and Ecosystems Health, College of Veterinary Medicine, Cornell University, Ithaca, NY 14853; ^d^Department of Biochemistry, Biophysics and Molecular Biology, University of Minnesota, Minneapolis, MN 55455; ^e^Department of Infectious Diseases, Mayo Clinic, Rochester, MN 55905; ^f^Institut für Tierhygiene und Öffentliches Veterinärwesen, Abteilung für Biochemie, Universität Leipzig, Leipzig 04103, Germany

**Keywords:** parvovirus, emergence, virology, antibodies, vaccine

## Abstract

Emergence of epidemic viruses in new hosts threatens both human and other animal populations, and often involves virus evolution to overcome barriers that normally prevent efficient infection and spread in that host. After transfer the separated viruses will evolve in parallel as they spread within the original and new hosts. Here we examine the details of a virus involved in such a host-jumping event, where we define the natural evolution of feline panleukopenia virus (FPV) over 60 y, clarify the origins of the new pandemic canine parvovirus (CPV) that arose in the 1970s, and compare the separate evolution of those viruses over 47 y in cats or dogs. Several live-attenuated FPV vaccine viruses originated from early-1960s isolates or were a recombinant of an early virus, and many sequences in databases proved to be vaccine-derived. The sequences of wild viruses showed that FPV-like strains evolved at less than one-third the rate observed for CPV in dogs, and the higher rate of CPV evolution has been consistent since 1979, when a genetic variant became widespread. The common ancestor of the CPV lineage was related to FPVs from Europe and contained several unique host-adaptive capsid changes associated with canine transferrin receptor type-1 binding. Although the FPV vaccine strains are around 60 y old, little selection for antigenic variation was observed. The distinct evolutionary patterns of these closely related viruses circulating for decades in different hosts emphasize the complex evolution associated with viral epidemic emergence and spread in endemic and new hosts.

## Significance

Comparing the evolution of a virus in its reservoir host with that seen in a new host will reveal the special circumstances that allow epidemic emergence. A feline parvovirus (FPV) that jumped to dogs in the mid-1970s formed canine parvovirus (CPV), which has circulated worldwide through today. The evolutionary rate of FPV in its original hosts was much lower than that of CPV in dogs, and the mutational patterns seen in the different hosts were distinct. Early CPV isolates differed from the ancestral FPV clade in several key host range mutations. These results highlight the complex biology associated with epidemic emergence, including host-specific rates of lineage evolution and complex origins of host-adaptive mutations.

Control of new viral diseases requires a clear understanding of their evolutionary dynamics allowing epidemic emergence, or of changes in antigenic structures that allow immune evasion (1). Viruses that emerge to cause epidemics or pandemics in new hosts represent significant threats to human and animal health (2). Our knowledge of how such viruses overcome the barriers that usually prevent emergences is incomplete (3–5), in particular the information about the ancestral virus and its evolutionary patterns in the original reservoir hosts (6). Comparing the evolution and biology of viruses in their original and new hosts can show how such emergence events have occurred, and how we might forestall similar outbreaks in the future (7).

Canine parvovirus (CPV) has been most recently classified as *Protoparvovirus carnivoran 1* (8), and its emergence from the feline panleukopenia virus (FPV) as a new virus in dogs occurred around 50 y ago (4, 9). Diseases in cats similar to those caused by FPV were reported around 1887 (10), and in the 1920s (11). In the late 1940s and early 1950s, a new disease seen among farmed mink was associated with an FPV-like parvovirus (12). FPV was first isolated in tissue culture in the early 1960s in the United Kingdom, and the sample was from a captive snow leopard (13). That virus was used to prepare attenuated viruses and since the 1970s attenuated FPV vaccine viruses have been included in the core vaccines recommended for all kittens (14).

In the mid-1970s a variant of FPV emerged in dogs that was named CPV type-2 (CPV-2) to distinguish it from the previously described minute virus of canines (MVC; now *Bocaparvovirus carnivoran 1*). Although first reported in many regions of the world during 1978 (15–18), CPV-2 strain was likely circulating in Western Europe for a few years before 1978, since anti-CPV antibodies were detected in sera collected from dogs in Europe between 1974 and 1977 (19–21). In other regions of the world, the first CPV-antibody positive sera were collected in early- to mid-1978 (22–24). Between 1979 and 1981 the original strain was replaced globally by a variant which contained several mutations and therefore was named CPV-2a (25, 26). In contrast to the CPV-2 strain, the CPV-2a strain had gained the feline host range (27, 28). Despite the feline host range of CPV-2a, most infections of cats since 1978 are due to FPV (29). Both FPV and CPV strains can naturally infect and cause disease in other hosts among the Order Carnivora (30).

The parvovirus capsid is a 26 nm diameter *T* = 1 icosahedron assembled from 60 copies of a combination of the overlapping VP1 and VP2 proteins (31). The small repeated exposed structure recognizes the host transferrin receptor type-1 (TfR), antibodies, and the sialic acid N-glycolylneuraminic acid (Neu5Gc) (32–35). The capsid packages a linear single-stranded DNA genome (ssDNA) of about 5,120 bases which encodes the nonstructural (NS) and the capsid or viral proteins (VP), including those from splice variant mRNAs or protease cleavage—NS1, NS2, VP1, VP2, and VP3 (36). Transmitted through a fecal-oral route, the virus is robust and can survive in the environment for weeks so it may be moved long distances on contaminated fomites (37).

Cell infection involves binding to the transferrin receptor type-1 (TfR) (38, 39). TfR is a homodimeric type-2 membrane glycoprotein which binds to iron-loaded transferrin (Tf), as well as other host proteins (ferritin and homeostatic iron regulator protein [also known as High FE2+ (HFE)], and it is a receptor for other pathogens (40, 41). The capsids of CPV and FPV bind TfR through a small surface of the TfR apical domain, as defined by cryoelectron microscopy (cryoEM) (42) and by mutational analysis of the capsid and receptor (43, 44). The canine host range of CPV-2 (and CPV-2a) is associated with a small number of capsid changes which allow binding to the canine TfR and infection of dog cells (45, 46). Some differences between FPV and CPV also altered antibody binding epitopes (32, 47–49) or the capsid interactions with the sialic acids (35, 50). FPV capsids do not bind the canine TfR or infect canine cells, due largely to the presence of a novel N-glycosylation in the canine TfR apical domain that falls within the capsid binding site (51). The mutations in CPV controlling canine host range allow that capsid to bind to the glycosylated canine TfR (47).

The parvovirus capsid rapidly induces protective antibodies (52) which recognize two dominant antigenic structures on the capsid surface (32–34, 53, 54). Many capsid mutations alter both antibody and TfR binding (33, 51, 55, 56), so that antigenicity and host range are coupled targets of selection in parvoviruses.

Previous studies examining the evolutionary patterns of FPV from cats or related hosts mostly examined the VP2 genes, and did not systematically identify the vaccine strains in the datasets (57–61). Recent studies have shown global distribution of FPV strains, and only a few mutations have spread widely—including the VP2 residue 91 Ala to Ser substitution (62, 63). We have recently defined 47 y of evolution of the full-genomes from natural infections of CPV in detail (64), and here we compare that to 60 y of FPV. We identify the origins of several live-attenuated vaccines in current use, reveal the closest relatives of CPV-2 among the FPV-like viruses, and reveal different rates and patterns of evolution between the two viral lineages during their decades of parallel spread.

## Materials and Methods

### FPV Sample Collection.

**Fig. 1.**
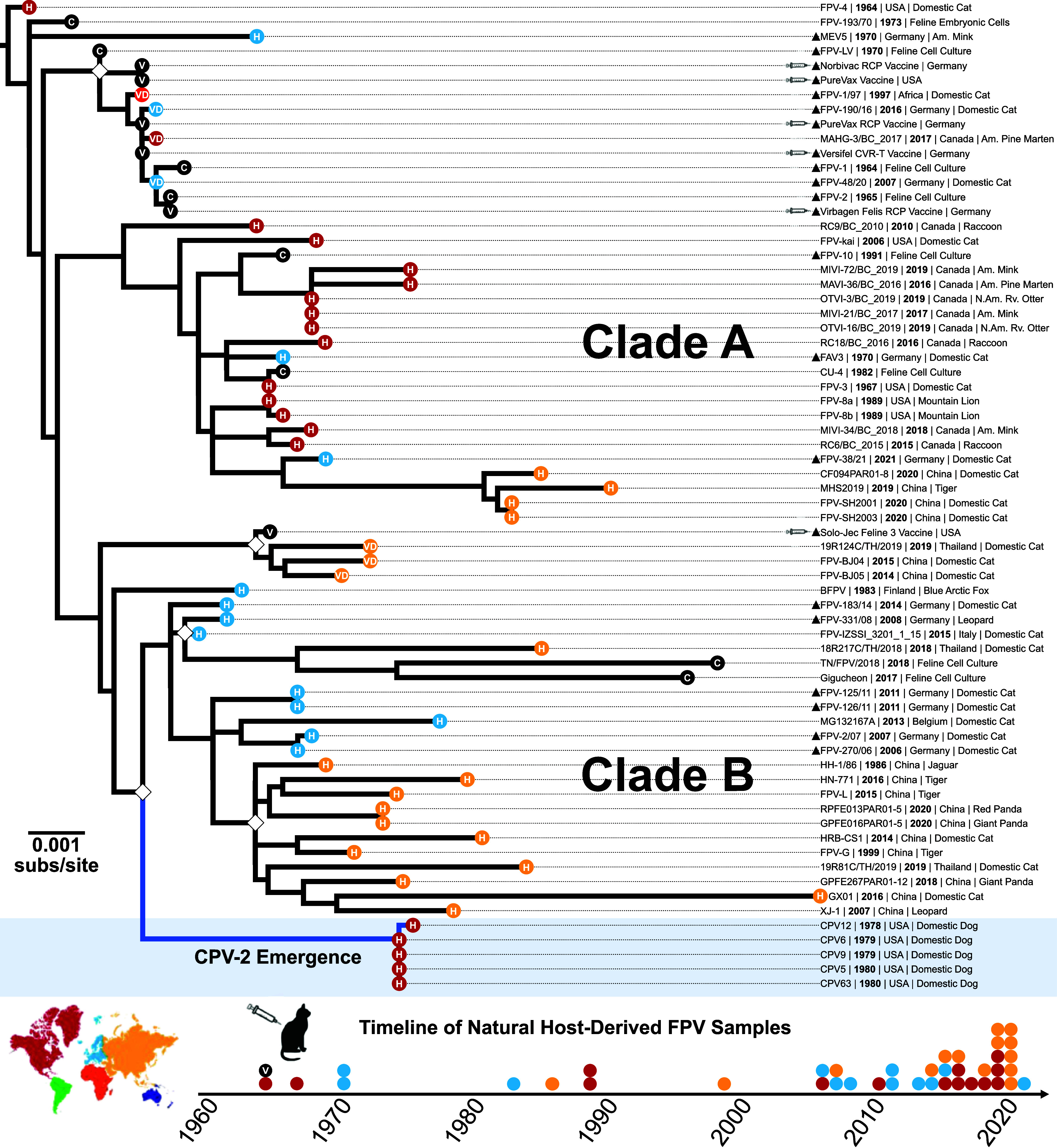
Evolutionary relationships among FPV full-genome sequences. ML tree of full-length FPV genomes (n = 63), in addition to an early CPV-2 emergent clade (n = 5). Newly isolated sequences are denoted with a triangle. Bootstrap support values of >90% are indicated with a white diamond at the nodes, scale represents nucleotide divergence. Branch tips are denoted with shapes colored by geographic origin (continent) and by sequence source (C = cell culture-adapted virus; H = host-derived virus, and V = vaccine-derived virus). A timeline of samples (below; colored by country as in the tree) shows the temporal and geographical origins of natural host-derived sequences analyzed.

Seventeen FPV-containing samples since 1964 were obtained from original host tissues or cell cultured isolates; these samples had been stored in our laboratory or obtained from other collections. The samples sequenced in this study are described in *SI Appendix*, Table S1 (and denoted in Fig. 1 with black triangles), along with other FPV full-genome sequences obtained from the GenBank NCBI database.

We also sequenced six commercially available live-attenuated FPV vaccine viruses obtained in 2022 and 2023. PureVax and Solo-Jec Feline 3 (Boehringer Ingelheim) were obtained in the United States. Virbagen Felis RCP (Virbac), Versifel CVR-T (Zoetis), PureVax RCP (Boehringer Ingelheim), and Nobivac RCP (Intervet International BV) were obtained in Germany.

### FPV Genome Amplification, DNA Library Preparation, and Sequencing.

Sequencing methods have been reported previously (64). Briefly, DNA isolated using the E.Z.N.A. Tissue DNA Kit (cat. no D3396-01) was used in PCR using the Q5 High Fidelity DNA Polymerase (New England BioLabs) as specified by the manufacturer. We amplified two overlapping genomic segments that covered the NS1 and VP1 reading frames using primers: 5’-CCGTTACTGACATTCGCTTCTTG-3’ and 5’-GAACTGCTCCATCACTCATTG-3’, and 5’-CATCCATCAACATCAAGACCAAC-3’ and 5’-CTTAACATATTCTAAGGGCAAACCAACCAA-3’. After purification 0.5 ng of each fragment was pooled together as input DNA (1 ng DNA total) to construct barcoded sequencing libraries with the Nextera XT DNA Library Preparation Kit (Illumina). Libraries were multiplexed and sequenced using MiSeq 2x250 Illumina sequencing. Raw read data can be found in NCBI Sequence Read Archive (SRA) under BioProject PRJNA1288508 (*SI Appendix*, Table S2).

### Illumina Read Processing, Analysis, and Bioinformatics.

Raw sequencing reads were trimmed using BBDuk (https://jgi.doe.gov/data-and-tools/software-tools/bbtools/bb-tools-user-guide/bbduk-guide/) to remove adaptors, PCR primers, and low-quality regions from reads. Reads were merged and mapped to an FPV reference sequence (consensus accession no. M38246) using Geneious Prime. Reads were error-corrected and normalized to 5,000-fold coverage per site using BBNorm (https://jgi.doe.gov/data-and-tools/software-tools/bbtools/bb-tools-user-guide/bbnorm-guide/). An average of ~5,000-fold coverage per site was obtained for a majority of the genome. Consensus genome sequence accession numbers are in *SI Appendix*, Tables S1 and S2.

### FPV Full-Genome and VP2 Phylogenetic Analyses.

We determined the evolutionary relationships among the full-genome consensus sequences (*n* = 63), including 22 sequences generated in this study (six from FPV vaccines), along with 41 FPV sequences from GenBank for which isolation location, date, and host information were available (*SI Appendix*, Table S1). To determine the most closely related FPV sequences to the early CPV emergence, we included sequences of 1978 and 1979 CPV-2 isolates in the phylogenetic analysis. Maximum likelihood (ML) phylogenetic analysis was performed using PhyML (65) or IQ-TREE (66), employing a general time-reversible (GTR) substitution model, gamma-distributed (Γ) rate variation among sites, and bootstrap resampling (1,000 replications). Trees were rooted to the earliest FPV sample available (FPV-4, 1964, accession no. EU659112).

We also examined trees prepared using midpoint rooting or rooted to an outgroup (porcine parvovirus, 64.1% identical, or Kilham rat parvovirus, 63.2% identical) to reveal the patterns shown.

Sequences of highly cell cultured viruses and identified vaccine virus genomes from the dataset were removed to allow analysis of the natural infection-derived sequences (*n* = 40) as both full-length genomes, and as NS1 and VP2 sequences separately. We also analyzed a larger dataset of FPV VP2 sequences from the GenBank NCBI database (*n* = 183). Comparison with the CPV natural genomes was done using a dataset of 212 sequences examined in a previous study of that virus (64).

### Mutation and Selection of FPV and Comparison with CPV.

Mutations in FPV genomes found in wildtype natural infections, vaccine virus, or highly cell-cultured samples were identified by comparison with the earliest FPV (FPV-4). Widespread polymorphisms in wildtype natural infections were counted if found in ≥10% of samples (at least 4 of 40). The rates of evolution of natural FPV (*n* = 40) and CPV (*n* = 212) genomes were determined using root-to-tip genetic distance by regression of each ML tree by date (year) in the TempEst v.1.5.3 software (67), and by the Bayesian Markov chain Monte Carlo (MCMC) approach run in BEAST v.1.10.4 (68). MCMC sampling was performed with a relaxed clock, a generalized time-reversible (GTR) substitution model with gamma distribution in four categories (Γ4), at a minimum of 100 million runs, with duplicates combined in Log Combiner v1.10.4. We confirmed statistical convergence with an effective sample size [ESS] ≥ 200 (consistent traces, removed burn-in at 10 per cent) in Tracer v1.7.2 (69). Parameter estimation is given as Highest Posterior Density (HPD) interval.

The relative numbers of nonsynonymous (dN) and synonymous (dS) nucleotide substitutions per site in NS1 and VP2 ORFs of FPV and CPV natural isolates were calculated using the SLAC (Single-Likelihood Ancestor Counting) method [Datamonkey package, https://datamonkey.org/ (70)]. A 95% CI was calculated for the mean dN/dS ratio and represented as error bars.

### Structural Analysis of FPV Capsid Mutations and Receptor or Antibody Binding Sites.

To model the homologous FPV mutations, the canine TfR structure was predicted using AlphaFold3 (71) and placed into the cryoEM density of the black-backed jackal (bbj) TfR in complex with the CPV-2 capsid using rigid-body fitting (EMDB ID: 20002) (PDB ID: 6OAS) (42). The bbjTfR structure was used since that is closely related to the canine TfR, but lacks the additional glycosylation found on that receptor, and was used in the TfR:capsid costructure. The N-glycan moieties [(Hex)2(HexNAc)1(NeuAc)1(Man)3(GlcNAc)2] on the canine TfR residue N384 were modeled into the TfR–CPV complex structure using the CHARMM-GUI Glycan Modeler (72). The effects of viral mutations on virus:ligand interactions were examined in the context of the cryo-EM structure by using Chimera X (73). Those included the bbjTfR in complex with the CPV-2 capsid (PDB ID: 6OAS) (42) and the structures of canine antibody Fab–capsid complexes (PDB IDs: 9E7W and 9E60) (74, 75).

### Statistical Analyses.

Data were analyzed using GraphPad Prism (GraphPad Software, Inc., La Jolla, CA).

## Results

### Sixty Years of FPV Evolution and Identification of Vaccine Viruses.

Here we reconstruct the evolutionary history of FPV and define the relationship between vaccine viruses and other isolates. The FPV sequences generated in this study, including those of live-attenuated vaccines widely used in the United States and Western Europe, allowed us to generate a maximum-likelihood (ML) tree of full-length FPV genomes (total n = 66), along with three CPV-2 genomes (Fig. 1). The tree topology of the major FPV clades (and position of the CPV-2) was consistent whether rooted to the earliest sample, using mid-point rooting, or rooting to distantly related (~64% identical) protoparvovirus outgroups. Most nodes had limited bootstrap support within the phylogeny, reflecting the fact that the sequences share >98.5% nucleotide identity overall. Most viruses were isolated from domestic cats, but some were collected from other susceptible hosts, including mink, raccoons, pine marten, river otter, pandas, and several other felids.

**Fig. 2.**
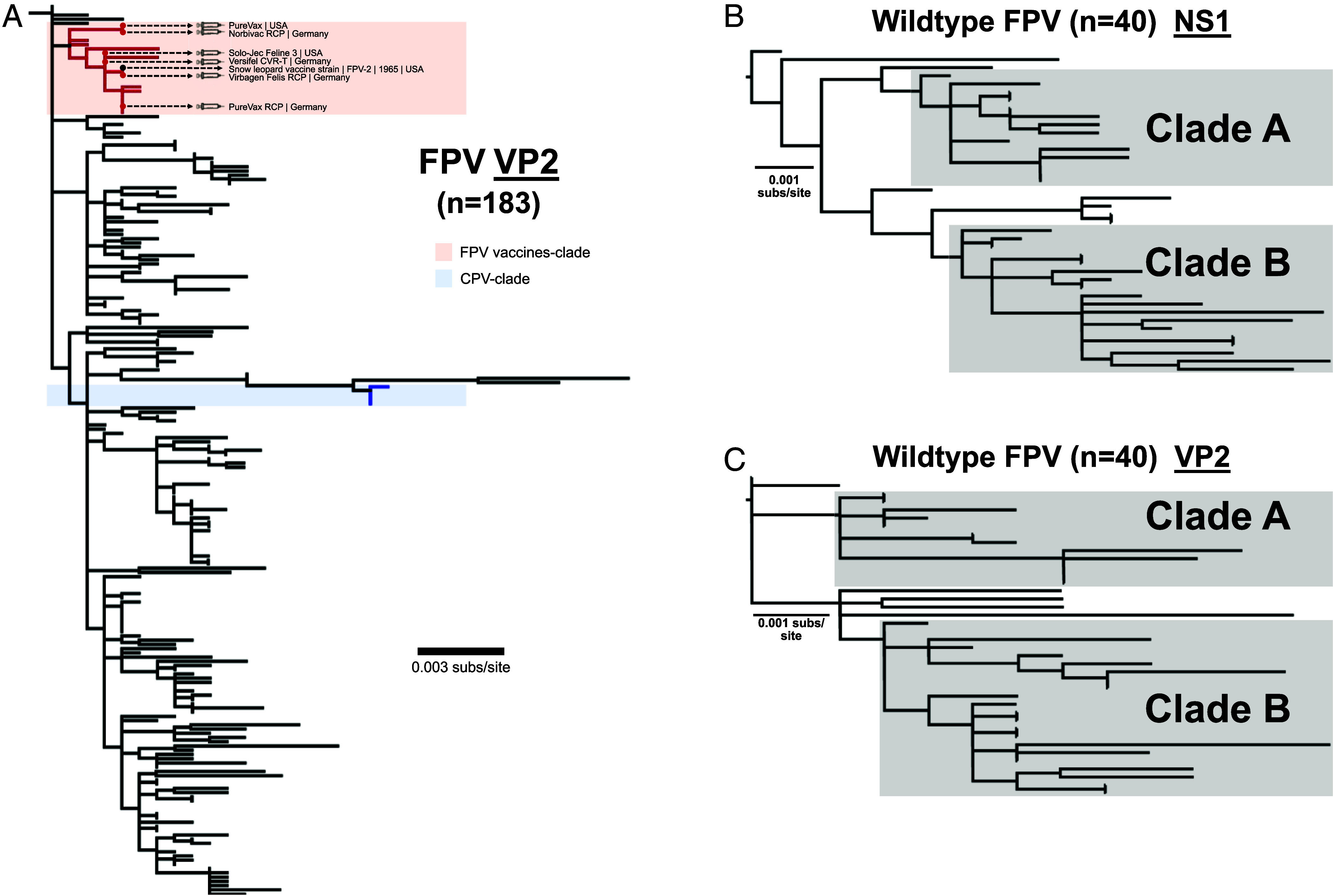
FPV vaccines have similar VP sequences and FPV natural sequences show convergent organization across genome and absence of natural recombination. (*A*) ML tree of expanded FPV VP2 sequences (n = 183) show the close relationship of all vaccine sequences in the capsid. ML trees of FPV natural sequences (n = 40) were generated for both NS1 (*B*) and VP2 (*C*) ORFs. Tree structure and clades match that seen with full-length genomes (Fig. 1).

Most vaccine virus sequences clustered with each other and with other early isolates (13). One vaccine virus (Solo-Jec Feline 3) grouped with more recent FPV samples in the full-genome phylogeny (Fig. 1). However, that was a recombinant, with a VP2 gene related to other vaccines and early 1960s isolates (Fig. 2*A*) and had a distinct NS1 sequence. A number of sequences reported as wildtype FPV strains clustered with the vaccine viral sequences and were, therefore, likely derived from vaccine viruses (Fig. 1).

After removing vaccine-derived and highly cell-culture passaged viruses, a collection of 40 FPV wildtype viruses was analyzed as complete genomes. The sequences fell into two distinct clades (Clades A and B) (Figs. 1 and 2), and while viruses showed a global distribution, North American isolates tended to fall within Clade A and those from Europe-Asia in Clade B. We also separately analyzed the NS1 and VP2 open reading frames, and those showed no obvious recombination lineages among that set of wildtype FPVs (Fig. 2 *B* and *C*).

### The Origin of CPV and Canine Adaptation.

**Fig. 3.**
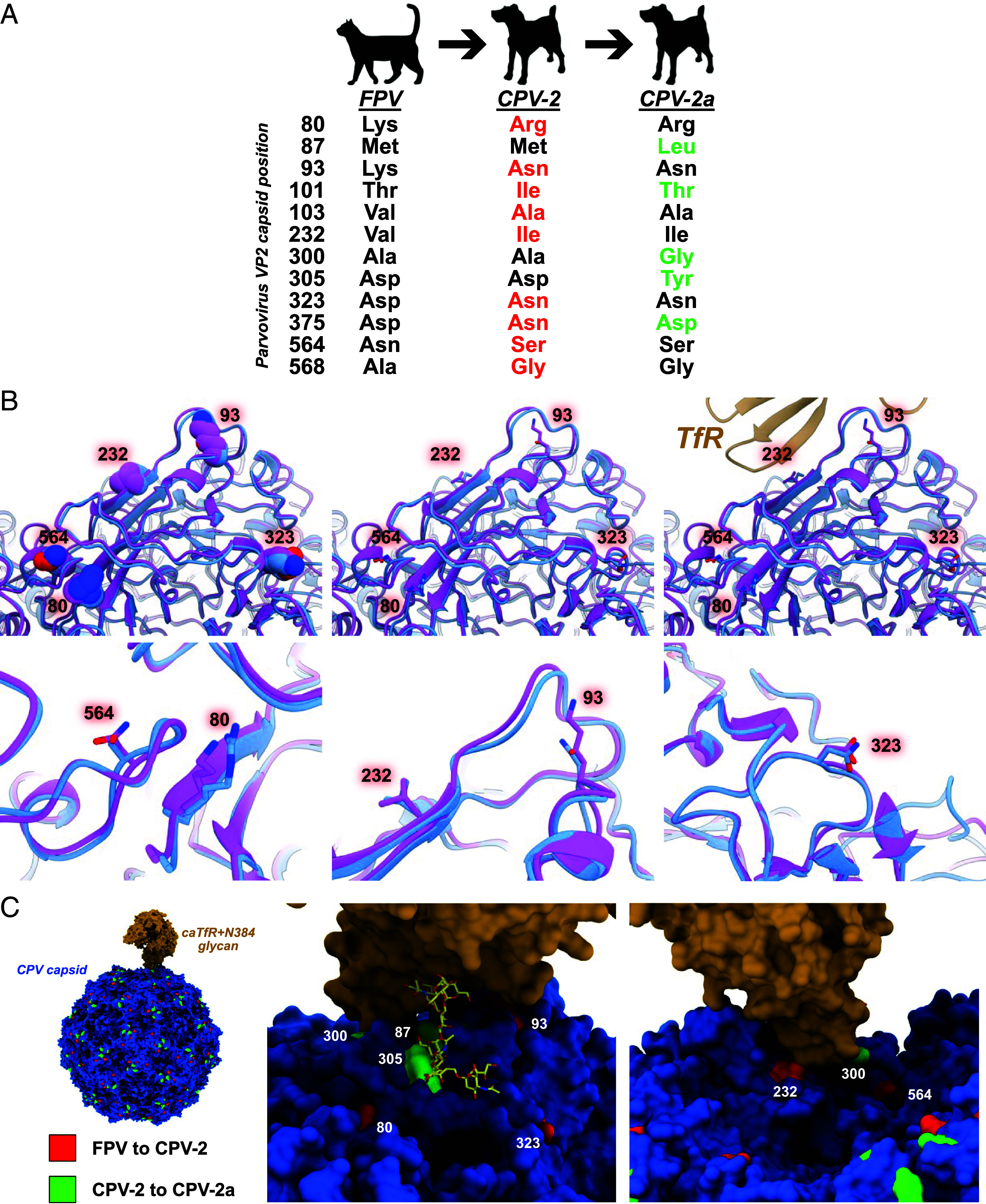
Molecular and structural evolution of parvovirus host shifts. Mutations on the branch leading to CPV-2 emergence. (*A*) Nonsynonymous VP2 mutations fixed during host shifts from likely FPV ancestor to earliest CPV-2 isolates (red) to CPV-2a pandemic (green). (*B*) Direct comparison of FPV-CPV-2 VP2 side chain changes at threefold spike of capsid, in relation to the TfR. (*C*) Structure of CPV capsid in interaction with canine TfR (including N-glycan structure). Positions in VP2 of mutations arising during shift to CPV-2 (red) and CPV-2a (green) are highlighted on the structure. All mutations present on the branch leading to emergent CPV-2 are listed in Table 1.

The branch leading to CPV-2 fell within a group of Clade B FPV sequences from Europe collected at various times after the emergence of CPV (Fig. 1) (25). That FPV—CPV branch included twelve nonsynonymous and ten synonymous substitutions across the genome (Table 1). Nine of the nonsynonymous substitutions fell within the capsid VP2 open reading frame (Fig. 3*A*). Differences in the VP2 capsid between the ancestral FPV and the earliest CPV-2 sequences included surface residues (80, 93, 232, 323, and 564) adjacent to the threefold spike, some of which influence the canine host range (Fig. 3*B*). The CPV-specific mutations (and subsequent CPV-2a replacement) within the capsid protein gene have clear effects on cell binding and infection using the nonglycosylated TfRs from cats, raccoons, and black-backed jackals, as well as the glycosylated canine TfR (Fig. 3*C*) (32, 42, 47, 49, 51).

**Table 1.** Alignment uncovered variations between consensus FPV lineage to earliest emergent CPV-2 genomes

Nucleotide	s/ns	ORF	Amino Acid
C743T	ns	NS1	T248I
T1224C	s	NS1	I408
A1479G	s	NS1	V493
var1620T	s	NS1	V540
G1926A	ns	NS1	M152V
var2002G	s	NS1	N/D668
C2110T	ns	VP1	L4F
A2753G	ns	VP2	K80R
A2760G	s	VP2	V82
A2793C	ns	VP2	K93N
C2816T	ns	VP2	T101I
T2822C	ns	VP2	V103A
G3208A	ns	VP2	V232I
T3213C	s	VP2	Y233
G3481A	ns	VP2	D323N
A3552G	s	VP2	E346
A3555G	s	VP2	A347
G3637A	ns	VP2	D375N
T3681C	s	VP2	T389
A4137C	s	VP2	A541
A4205G	ns	VP2	N564S
C4217G	ns	VP2	A568G

### Evolutionary Rates of FPV and CPV.

**Fig. 4.**
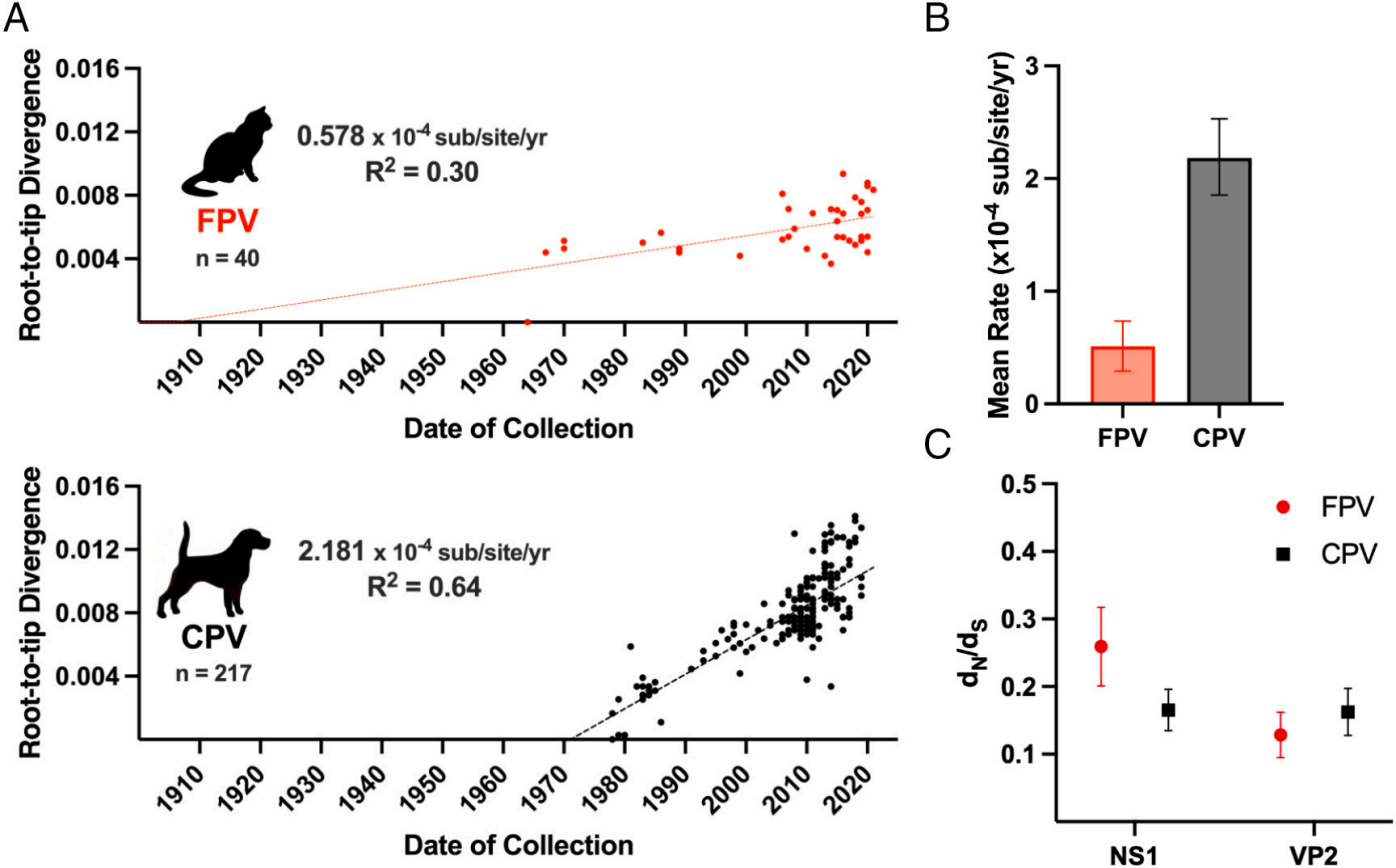
FPV and CPV diverge in evolutionary rates in each host. (*A*) Divergence rates of FPV (*n* = 40) and CPV (*n* = 217) among recent natural sequences were generated from ML trees analyzed using root-to-tip regression in TempEst. (*B*) Substitution rates were calculated following a Coalescence Model analysis of natural FPV and CPV sequences. (*C*) Calculated dN/dS ratios of respective gene ORFs among natural FPV and CPV sequences (±SEM).

Examining the 47 y of parallel spread in cats and dogs since 1978 showed significant differences in the rates of evolution of the virus in the original versus the new host. Substitution rates of natural isolates of FPV and CPV (*n* = 40 and *n* = 212, respectively) were estimated using both root-to-tip divergence (Fig. 4*A*) and a Bayesian Coalescence Model (Fig. 4*B*). Those approaches showed FPV variation rates of 5.78 × 10^−5^ and 5.11 × 10^−5^ [HPD 2.03 × 10^−5^ –7.369 × 10^−5^] substitutions/site/year, and CPV rates of 2.181 × 10^−4^ and 2.184 × 10^−4^ [HPD 1.855 × 10^−4^ – 2.532 × 10^−4^] substitutions/site/year. The CPV divergence rate was therefore 3 to 4 times that seen for FPV (Fig. 4). In addition, the evolutionary rates of FPV and CPV lineages each appeared relatively constant during the entire periods analyzed, with a key exception being seen in the first 2 y of CPV spread, where several mutations were all fixed together in the ancestor of the CPV-2a lineage (Fig. 3).

### Patterns of Variation in FPV Genomes and Comparison to CPV.

**Fig. 5.**
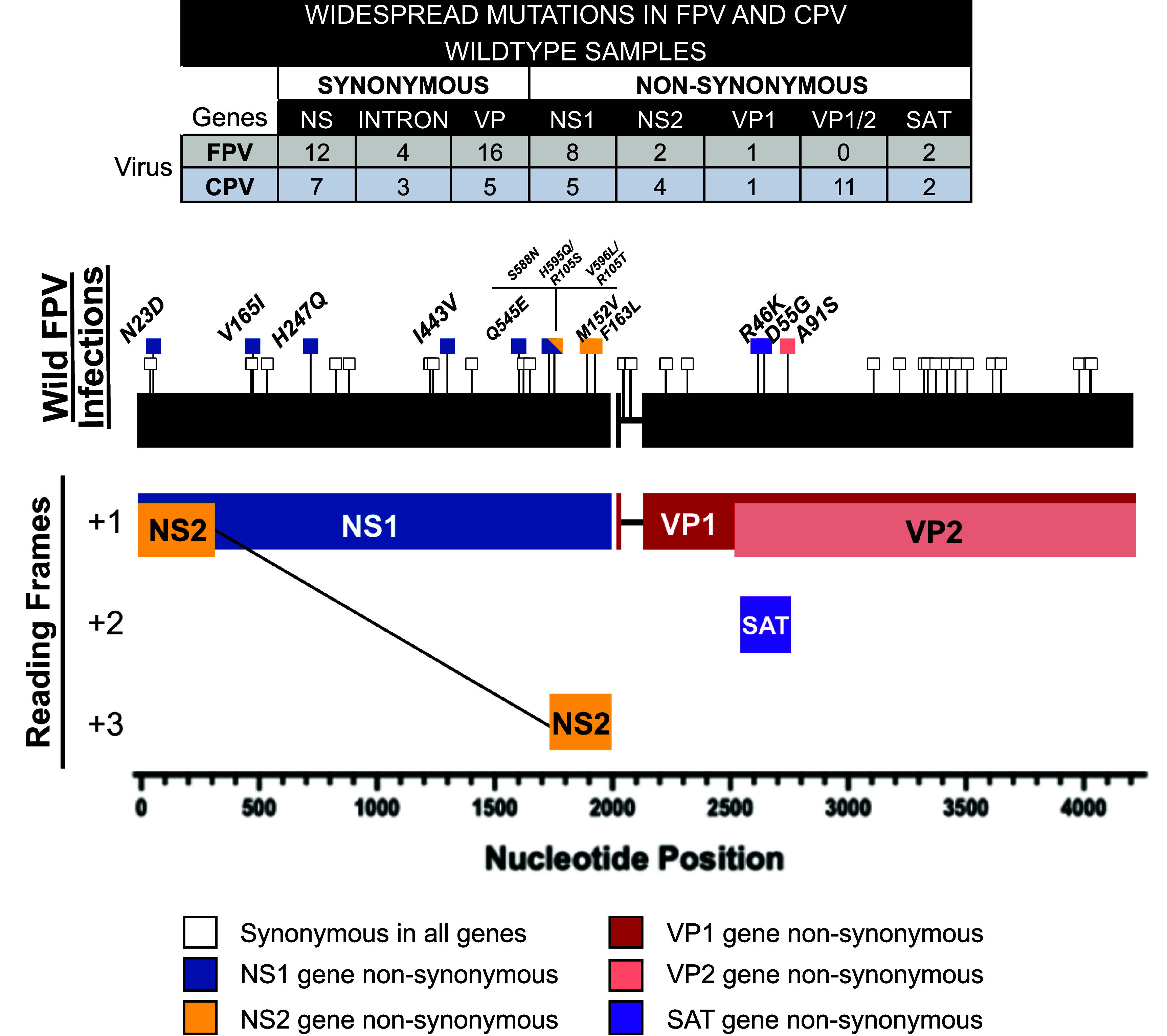
Mutational variation in wildtype FPV genomes. FPV genomes were aligned and categorized by source (wildtype, cell culture, vaccines). Widespread polymorphic variants among wildtype natural infections (≥10%) were determined among FPV (n = 40) and CPV (n = 217) samples. Mutation counts by type and ORF position are tabled; a full listing of widespread variants is found in *SI Appendix*, Table S3. FPV mutations are visualized on the genome using pins (nonsynonymous = colored squares, synonymous = white squares). Nucleotide numbering begins at the first coding site of the NS1/2 gene, and amino acid numbering is based on the translational start site of each respective gene.

Comparing the ratios of nonsynonymous to synonymous (dN/dS) mutations among wildtype FPV and CPV sequences over their circulation showed minimal selection and no significant differences between each virus in their respective hosts. The ratio for NS1 genes was somewhat greater for FPV (0.26 ± 0.06) than seen for CPV (0.17 ± 0.03), while those for the VP2 genes were similar (0.13 ± 0.03 versus 0.16 ± 0.03, respectively) (Fig. 4*C*). There were 45 widespread mutations or polymorphisms among the FPV genomes, including 32 that were synonymous and 13 that were nonsynonymous (Fig. 5 and *SI Appendix*, Table S3). Of the synonymous mutations, 12 fell within the nonstructural genes (NS) and 16 within the VP coding region. For the 13 nonsynonymous mutations, 6 were found in the NS1 gene, 2 were found to impact both the NS1 and NS2 overlapping reading frames, 2 were in the NS2 gene region alone, 1 was found in the VP2 gene, and 2 were in the small alternatively translated (SAT) alternate open reading frame within the VP2 gene. Compared to CPV the FPV sequences acquired more synonymous and fewer nonsynonymous changes (Fig. 5) (64). Many (12 of 23) of the nonsynonymous substitutions in CPV were in the VP1/2 gene, compared to one of 13 in the FPV VP1/2 (Fig. 5). The functions of most mutations within the FPV NS1, NS2, and SAT genes are not known, but host-specific effects may be possible, as these proteins interact with host-derived cellular proteins (76–81).

FPV samples collected from hosts other than cats showed few specific sequence patterns (57, 59, 82, 83), apart from a VP2 Gly229Glu (G229E) mutation in giant panda isolates (84, 85).

### Capsid Mutations, FPV Vaccines, and Antigenic Variation.

**Fig. 6.**
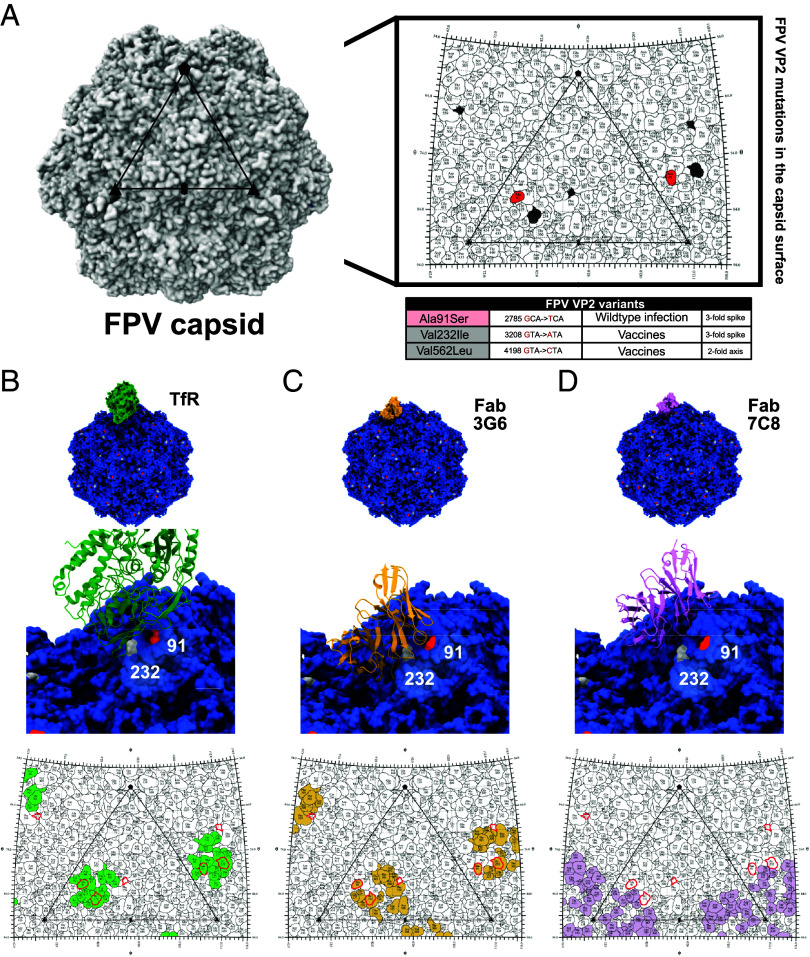
Widespread FPV VP2 variants and their relative position in the virus capsid surface in context with TfR and antibody footprints. (*A*) Reconstruction of the FPV capsid, showing axes of symmetry (oval = twofold axis, triangle = threefold axis, and pentagon = fivefold axes), and a roadmap of a single icosahedral asymmetric unit (PDB ID: 1C8G). The table below the roadmap identifies the widespread variants shared among wildtype infection samples (red) or majority of vaccine strains (gray). (*B*) Reconstruction of the high-resolution structure of the parvovirus capsid in complex with TfR (green, PDB ID: 6OAS) with corresponding roadmap where variants are outlined in red relative to the TfR footprint (green). (*C*) Reconstruction of the FPV capsid in complex with a canine-derived monoclonal antibody as Fab: 3G6 (yellow, PDB ID: 9E7W) with corresponding roadmap where variants are outlined in red relative to the Fab3G6 footprint (yellow). (*D*) Reconstruction of the FPV capsid in complex with a canine-derived monoclonal antibody as Fab: 7C8 (purple, PDB ID: 9E60) with corresponding roadmap where variants are outlined in red relative to the Fab7C8 footprint (purple).

The only consistent variant that became widespread among wildtype FPVs since the 1960s was VP2 Ala91Ser (A91S). That is exposed in the threefold spike (Fig. 6*A*) and falls within the TfR footprint (Fig. 6*B*) and the binding site of canine antibody Fab2C5 (Fig. 6*C*) and is adjacent to antibody Fab7C8 footprint (Fig. 6*D*).

Live-attenuated FPV vaccines analyzed were all closely related to early FPV isolates, including the snow leopard strain from around 1964 (FPV-2) (Fig. 1). The vaccine strains often have unique or shared nonsynonymous changes in NS1 and NS2. Two VP2 nonsynonymous changes were shared among the majority of the vaccine strains, including the Val232Ile (V232I) change on the threefold spike and Val562Leu (V562L) near the twofold axis—and both were exposed on the capsid surface (Fig. 6*A*). Residue VP2 232 fell within the TfR binding footprint, while VP2 residue 562 was immediately adjacent to it (Fig. 6*B*). Similarly, the FPV vaccine variant at residue 232 is within or close to both canine antibody binding sites (54), while VP2 residue 562 is adjacent to one of these sites (Fig. 6 *C* and *D*).

## Discussion

Here we directly compare two parvoviruses before and after the emergence of one of those to cause a pandemic that has continued for around 50 y. We show that the original FPVs in cats and related carnivore hosts evolve slowly with few nonsynonymous mutations, that they are distributed among two clades that are partially geographically structured, and that the single branch that contained several host-adaptive coding changes gave rise to the ancestor of the CPV lineage. This highlights the complex processes required for the virus to successfully overcome a host-range barrier and to spread widely in its new host.

An aspect of our analysis is that the FPV genomes were derived from natural infections over 60 y, allowing us to exclude sequences from the widely used attenuated vaccine viruses. This detailed understanding of the FPV and CPV sequence evolution is paired with the structures and functions of their respective capsids, their interactions with TfRs from different hosts, as well as with natural postinfection host antibodies.

### FPV Strains Show Distinct Patterns of Variation Compared to CPV.

Over the 47 y of parallel spread in different hosts the FPV sequences evolved at around 0.5 × 10^−4^ subs/site/year, compared to 2.2 × 10^−4^ subs/site/year for CPV (Fig. 4 *A* and *B*). The relatively higher evolutionary rate of CPV sequences could only be resolved most clearly throughout the 47 y since it spread worldwide. It is unclear how, and by what mechanism, the evolution of CPV progressed comparatively during the earlier posttransfer period (1979 to 1989) and later (1989 to 2024). Both the CPV emergence from the FPV-clade and the further emergence of the CPV-2a lineage involved many observed mutations, including several host-adaptive changes, in a relatively short order of time (25, 26, 48). Given the generally low rates of evolution in ssDNA parvoviruses, it is unclear how those groups of mutations arose and were fixed by gradual evolution.

Explanations for the overall faster evolution of the CPV lineage may include multiple nonexclusionary hypotheses. Given the immune naivete of dogs and the worldwide spread of the virus as a pandemic of dogs, an early expansive population size is likely, as well as continuing host or antigenic selection. Greater genetic drift resulting from different epidemiological properties in dogs can be considered. Observations of host-specific rate variation are common (86), and have also been measured for influenza A and SARS-CoV-2 viruses (87, 88). The involvement of additional hosts beyond cats or dogs is another hypothesis—for example, apparent partial intermediate viruses have been detected in raccoons, which may suggest pathways for the acquisition of mutational combinations that would be unfit in either cats or dogs but may have facilitated new potential genotypes (89).

Little parallel (convergent) evolution was observed between the FPV and CPV lineages since they diverged in 1978, with only VP2 V232I being seen among both FPV and CPV sequences (64), indicating there were different selective pressures or genetic drift in each host. The baseline evolutionary rate seen for carnivore parvoviruses (as exhibited by the FPV rate) in this study is similar to that determined for human B19 parvoviruses using combinations of contemporary- and ancient DNA-derived sequences, which were calculated to be 1 to 3 × 10^−5^ subs/site/year (90, 91), both examining well-adapted parvoviruses spreading in their normal host.

### Changes Selected in Alternative Hosts.

Cat and dog populations vastly outnumber those of other carnivore hosts for these viruses, and so we expect that most FPV and CPV evolution occurs in those animals. No host-specific variation was identified among FPVs collected from snow leopards, wild cats, minks, raccoons, or bobcats, while the variation of VP2 residue 299 (Ala to Glu) may be host-adaptive for giant pandas (84, 85). In contrast, CPVs from noncanine hosts often show variations in capsid structures associated with TfR binding (89, 92), likely needed for efficient binding and infection using the nonglycosylated TfRs in those hosts (32, 47, 51).

### Sixty-Year-Old FPV Vaccine Strains Select Little Antigenic Variation.

The FPV live-attenuated vaccines examined here were all derived from one or a few 60-year-old isolates, and those are included in core feline vaccines around the world (14). The natural evolution of FPV included only one or two variations within the two main capsid epitopes, suggesting little or no antigenic selection of the virus infecting cats. In contrast, several coding changes and antigenic variants of CPV have been observed, although there is no clear evidence of immune escape (93, 94). Escape from individual monoclonal antibodies occurs readily in parvoviruses in in vitro studies (95). However, although the host polyclonal antibody repertoire against the CPV (and likely FPV capsid) is limited, that appears sufficient to block infection and circulation of all strains of the virus in its animal hosts (96, 97). The FPV (and CPV) therefore is similar to other viruses where vaccines have retained their efficacy for decades with little immune evasion arising, such as smallpox, measles, and rinderpest viruses (98–100).

### All These Viruses May Share a Recent Common Ancestor, and CPV-2 Is Most Closely Related to European FPV Strains.

Phylogenetic and rate analysis of all the FPV-derived (including CPV) sequences suggests that those viruses may share a common ancestor that existed in the late 19th century. The first reports of feline ataxia due to cerebellar hypoplasia in two kittens were from 1887 (10), while other FPV-associated diseases in cats and raccoons were reported in the 1920s and 1930s (11, 101). However, backextrapolation of the sequences collected since the 1960s may not be accurate, and these viruses may have been circulating for much longer. For comparison, while recent human B19 parvovirus sequences also appeared to converge upon a common ancestor in the 19th century (86, 102), ancient DNA in human remains up to ~7,000 y old showed that similar strains existed at that time (90, 91). Sequences of FPVs derived from ancient cat samples would reveal a more accurate rate if those could be found.

The CPV-2 strain was recognized, and the first viral sequences were collected, when it spread worldwide in 1978 (9, 17, 18). However, serological testing showed that the virus was circulating in dogs in Western Europe between 1974 and 1978 (19–21), and the ancestor of the CPV lineage fell within a group of FPVs from Europe (Figs. 1 and 2). As those European FPV isolates were collected after 1978 (between 2006 and 2016), they would be descendants of viruses similar to the CPV ancestor. The true ancestor may still be sought among archival tissues collected from dogs, cats, or related hosts in Europe or Eurasia during the early to mid-1970s. This analysis also rules out the possibility that CPV emerged from an FPV vaccine (Fig. 1) (103, 104).

### Evolution of the Canine Host Range.

While dogs would have been consistently exposed to FPV, CPV is only known to have emerged once to cause an epidemic. The host barrier of dogs was high, with several host-adaptive changes being present in the long branch between FPV and CPV-2—including VP2 residues 93, 103, 323, and 375 (Fig. 3*A*) (32). While some variation of those sites has been reported [e.g., VP2 93 Lys in a CPV background (60), VP2 93 Asn in an FPV background (61)] those are rare exceptions. Canine host range resulted from the virus adapting the capsid contacts with the canine TfR, including both protein–protein and protein–glycan interactions (38, 45, 51, 105), and key mutations were associated with small changes in capsid structure, which together allowed them to engage the canine TfR to allow efficient entry and infection (Fig. 3 *B* and *C*) (42, 55).

The slow evolution of FPV in its normal hosts indicates that acquiring several canine host range mutations involved a different evolutionary process. Parvoviruses differ from the RNA viruses that are often suggested to be most likely to emerge due to their high mutation rates. The ssDNA parvovirus genomes are replicated by host cell DNA polymerase during cellular S-phase (most commonly pol δ), likely without the error-correcting mechanisms of host genome replication (106), and their error rates of replication are likely about 10-fold lower than most RNA viruses. Although polymorphisms are rarely detected within deep-sequenced parvovirus DNA samples (64), mutant genomes are being generated at a level where they can be quickly selected—including by host cells or neutralizing antibodies (64, 95). It is likely that the several canine-adaptive mutations found in CPV-2 (and then in CPV-2a) all arose through normal processes of mutation, but with strong selection for the use of the canine TfR to bind and infect dogs.

### Summary.

Comparing the evolution of a virus in its reservoir, the process of host jumping, and the posttransfer evolution shows that viral emergence in a new host required a special event that allowed the rapid acquisition of several mutations to overcome the high host range barrier posed by dogs. Similar to other examples of evolution involving punctuated equilibrium, those mutations would not have arisen and been selected in the normal course of evolution of the FPV but were likely due to strong selection within dogs (possibly with involvement of other intermediate hosts). After pandemic emergence, the higher evolutionary rate and increase of nonsynonymous changes in CPV suggests that there is continuing selection in dogs, even decades after the host jump. These general features are similar to those seen for some other emerging viruses such as HIV-1 and influenza virus, where many different mutations and factors were required for the virus to successfully become a pandemic pathogen in a new host (6).

## Supplementary Material

Appendix 01 (PDF)

## Data Availability

DNA sequences data have been deposited in Genbank, NCBI Sequence Read Archive (SRA) (PRJNA1288508) (107).

## References

[1] O. G. Pybus, A. Rambaut, Evolutionary analysis of the dynamics of viral infectious disease. Nat. Rev. Genet. **10**, 540–550 (2009). 19564871 10.1038/nrg2583 PMC7097015

[2] D. M. Morens, P. Daszak, H. Markel, J. K. Taubenberger, Pandemic COVID-19 joins history’s pandemic legion. mBio **11**, e00812-20 (2020). 32471830 10.1128/mBio.00812-20 PMC7267883

[3] R. K. Plowright , Pathways to zoonotic spillover. Nat. Rev. Microbiol. **15**, 502–510 (2017). 28555073 10.1038/nrmicro.2017.45 PMC5791534

[4] C. R. Parrish , Cross-species virus transmission and the emergence of new epidemic diseases. Microbiol. Mol. Biol. Rev. **72**, 457–470 (2008). 18772285 10.1128/MMBR.00004-08 PMC2546865

[5] E. C. Holmes, The ecology of viral emergence. Annu. Rev. Virol. **9**, 173–192 (2022). 35704744 10.1146/annurev-virology-100120-015057

[6] B. R. Wasik , Onward transmission of viruses: How do viruses emerge to cause epidemics after spillover? Philos. Trans. R. Soc. Lond. B. Biol. Sci. **374**, 20190017 (2019). 31401954 10.1098/rstb.2019.0017 PMC6711314

[7] J. L. Geoghegan, E. C. Holmes, Predicting virus emergence amid evolutionary noise. Open Biol. **7**, 170189 (2017). 29070612 10.1098/rsob.170189 PMC5666085

[8] F. M. Zerbini , Changes to virus taxonomy and the ICTV Statutes ratified by the International Committee on Taxonomy of Viruses (2023). Arch. Virol. **168**, 175 (2023). 37296227 10.1007/s00705-023-05797-4 PMC10861154

[9] C. R. Parrish, Host range relationships and the evolution of canine parvovirus. Vet. Microbiol. **69**, 29–40 (1999). 10515266 10.1016/s0378-1135(99)00084-x

[10] W. Herringham, F. Andrewes, Two cases of cerebellar disease in cats, with staggering. St. Barth. Hosp. Rep. **24**, 241–248 (1888).

[11] J. Verge, N. Christoforoni, La gastroenterite infectieuse des chats; est-elle due à un virus filtrable. C. R. Seances Soc. Biol. Fil. **99**, 312 (1928).

[12] R. H. Johnson, J. G. Cruickshank, Problems in classification of feline panleucopaenia virus. Nature **212**, 622–623 (1966). 4961647 10.1038/212622a0

[13] R. H. Johnson, Isolation of a virus from a condition stimulating feline panleukopenia in a leopard. Vet. Rec. **76**, 1008–1013 (1964).

[14] A. E. Stone , 2020 AAHA/AAFP feline vaccination guidelines. J. Feline Med. Surg. **22**, 813–830 (2020). 32845224 10.1177/1098612X20941784 PMC11135662

[15] D. T. Nelson, S. L. Eustis, J. P. McAdaragh, I. Stotz, Lesions of spontaneous canine viral enteritis. Vet. Pathol. **16**, 680–686 (1979). 505892 10.1177/030098587901600606

[16] W. F. Robinson, G. E. Wilcox, R. L. Flower, Canine parvoviral disease: Experimental reproduction of the enteric form with a parvovirus isolated from a case of myocarditis. Vet. Pathol. **17**, 589–599 (1980). 7404970 10.1177/030098588001700508

[17] M. J. Appel, B. J. Cooper, H. Greisen, F. Scott, L. E. Carmichael, Canine viral enteritis. I. Status report on corona- and parvo-like viral enteritides. Cornell Vet. **69**, 123–133 (1979). 223812

[18] M. J. Appel, F. W. Scott, L. E. Carmichael, Isolation and immunization studies of a canine parvo-like virus from dogs with haemorrhagic enteritis. Vet. Rec. **105**, 156–159 (1979). 516347 10.1136/vr.105.8.156

[19] A. Schwers, P.-P. Pastoret, G. Burtonboy, E. Thiry, Fréquence en Belgique de l’infection a Parvovirus chez le chien, avant et après l’observation des premiers cas cliniques. Ann. Méd. Vét. **123**, 561–566 (1979).

[20] A. D. Osterhaus, G. A. Drost, R. M. Wirahadiredja, T. S. van den Ingh, Canine viral enteritis: Prevalence of parvo-, corona- and rotavirus infections in dogs in the Netherlands. Vet. Q. **2**, 181–190 (1980). 22039978 10.1080/01652176.1980.9693779

[21] G. Koptopoulos, O. Papadopoulos, M. Papanastasopoulou, H. J. Cornwell, Presence of antibody cross-reacting with canine parvovirus in the sera of dogs from Greece. Vet. Rec. **118**, 332–333 (1986). 3705371 10.1136/vr.118.12.332

[22] S. T. Walker, C. P. Feilen, M. Sabine, D. N. Love, R. F. Jones, A serological survey of canine parvovirus infection in New South Wales, Australia. Vet. Rec. **106**, 324–325 (1980). 7376383 10.1136/vr.106.15.324

[23] L. E. Carmichael, J. C. Joubert, R. V. Pollock, Hemagglutination by canine parvovirus: Serologic studies and diagnostic applications. Am. J. Vet. Res. **41**, 784–791 (1980). 6250432

[24] S. Mohri, S. Handa, T. Wada, S. Tokiyoshi, Sero-epidemiologic survey on canine parvovirus infection. Nihon Juigaku Zasshi. **44**, 543–545 (1982). 7132022 10.1292/jvms1939.44.543

[25] C. R. Parrish , The global spread and replacement of canine parvovirus strains. J. Gen. Virol. **69**, 1111–1116 (1988). 2836554 10.1099/0022-1317-69-5-1111

[26] C. R. Parrish, P. H. O’Connell, J. F. Evermann, L. E. Carmichael, Natural variation of canine parvovirus. Science **230**, 1046–1048 (1985). 4059921 10.1126/science.4059921

[27] U. Truyen, J. F. Evermann, E. Vieler, C. R. Parrish, Evolution of canine parvovirus involved loss and gain of feline host range. Virology **215**, 186–189 (1996). 8560765 10.1006/viro.1996.0021

[28] Y. Ikeda, K. Nakamura, T. Miyazawa, E. Takahashi, M. Mochizuki, Feline host range of canine parvovirus: Recent emergence of new antigenic types in cats. Emerg. Infect. Dis. **8**, 341–346 (2002). 11971764 10.3201/eid0804.010228 PMC2730235

[29] B. Stuetzer, K. Hartmann, Feline parvovirus infection and associated diseases. Vet. J. **201**, 150–155 (2014). 24923754 10.1016/j.tvjl.2014.05.027

[30] C. R. Parrish, Emergence, natural history, and variation of canine, mink, and feline parvoviruses. Adv. Virus Res. **38**, 403–450 (1990). 2171302 10.1016/S0065-3527(08)60867-2 PMC7131698

[31] J. Tsao , The three-dimensional structure of canine parvovirus and its functional implications. Science **251**, 1456–1464 (1991). 2006420 10.1126/science.2006420

[32] S. F. Chang, J. Y. Sgro, C. R. Parrish, Multiple amino acids in the capsid structure of canine parvovirus coordinately determine the canine host range and specific antigenic and hemagglutination properties. J. Virol. **66**, 6858–6867 (1992). 1331498 10.1128/jvi.66.12.6858-6867.1992 PMC240290

[33] M. L. Strassheim, A. Gruenberg, P. Veijalainen, J. Y. Sgro, C. R. Parrish, Two dominant neutralizing antigenic determinants of canine parvovirus are found on the threefold spike of the virus capsid. Virology **198**, 175–184 (1994). 8259653 10.1006/viro.1994.1020

[34] S. Hafenstein , Structural comparison of different antibodies interacting with parvovirus capsids. J. Virol. **83**, 5556–5566 (2009). 19321620 10.1128/JVI.02532-08 PMC2681957

[35] J. Löfling, S. M. Lyi, C. R. Parrish, A. Varki, Canine and feline parvoviruses preferentially recognize the non-human cell surface sialic acid N-glycolylneuraminic acid. Virology **440**, 89–96 (2013). 23497940 10.1016/j.virol.2013.02.009 PMC3634669

[36] R. A. López-Astacio, O. F. Adu, H. Lee, S. L. Hafenstein, C. R. Parrish, The structures and functions of parvovirus capsids and missing pieces: The viral DNA and its packaging, asymmetrical features, nonprotein components, and receptor or antibody binding and interactions. J. Virol. **97**, e0016123 (2023). 37367301 10.1128/jvi.00161-23 PMC10373561

[37] A. Cavalli , In vitro virucidal activity of sodium hypochlorite against canine parvovirus type 2. Epidemiol. Infect. **146**, 2010–2013 (2018). 30178730 10.1017/S0950268818002431 PMC6452984

[38] J. S. Parker, W. J. Murphy, D. Wang, S. J. O’Brien, C. R. Parrish, Canine and feline parvoviruses can use human or feline transferrin receptors to bind, enter, and infect cells. J. Virol. **75**, 3896–3902 (2001). 11264378 10.1128/JVI.75.8.3896-3902.2001 PMC114880

[39] K. Hueffer, L. M. Palermo, C. R. Parrish, Parvovirus infection of cells by using variants of the feline transferrin receptor altering clathrin-mediated endocytosis, membrane domain localization, and capsid-binding domains. J. Virol. **78**, 5601–5611 (2004). 15140957 10.1128/JVI.78.11.5601-5611.2004 PMC415789

[40] B. E. Eckenroth, A. N. Steere, N. D. Chasteen, S. J. Everse, A. B. Mason, How the binding of human transferrin primes the transferrin receptor potentiating iron release at endosomal pH. Proc. Natl. Acad. Sci. U.S.A. **108**, 13089–13094 (2011). 21788477 10.1073/pnas.1105786108 PMC3156180

[41] C. Testi, A. Boffi, L. C. Montemiglio, Structural analysis of the transferrin receptor multifaceted ligand(s) interface. Biophys. Chem. **254**, 106242 (2019). 31419721 10.1016/j.bpc.2019.106242

[42] H. Lee , Transferrin receptor binds virus capsid with dynamic motion. Proc. Natl. Acad. Sci. U.S.A. **116**, 20462–20471 (2019). 31548398 10.1073/pnas.1904918116 PMC6789729

[43] L. B. Goodman , Binding site on the transferrin receptor for the parvovirus capsid and effects of altered affinity on cell uptake and infection. J. Virol. **84**, 4969–4978 (2010). 20200243 10.1128/JVI.02623-09 PMC2863798

[44] L. M. Palermo, K. Hueffer, C. R. Parrish, Residues in the apical domain of the feline and canine transferrin receptors control host-specific binding and cell infection of canine and feline parvoviruses. J. Virol. **77**, 8915–8923 (2003). 12885908 10.1128/JVI.77.16.8915-8923.2003 PMC167234

[45] K. M. Stucker , The role of evolutionary intermediates in the host adaptation of canine parvovirus. J. Virol. **86**, 1514–1521 (2012). 22114336 10.1128/JVI.06222-11 PMC3264339

[46] K. Hueffer, L. Govindasamy, M. Agbandje-McKenna, C. R. Parrish, Combinations of two capsid regions controlling canine host range determine canine transferrin receptor binding by canine and feline parvoviruses. J. Virol. **77**, 10099–10105 (2003). 12941920 10.1128/JVI.77.18.10099-10105.2003 PMC224579

[47] K. Hueffer , The natural host range shift and subsequent evolution of canine parvovirus resulted from virus-specific binding to the canine transferrin receptor. J. Virol. **77**, 1718–1726 (2003). 12525605 10.1128/JVI.77.3.1718-1726.2003 PMC140992

[48] L. A. Shackelton, C. R. Parrish, U. Truyen, E. C. Holmes, High rate of viral evolution associated with the emergence of carnivore parvovirus. Proc. Natl. Acad. Sci. U.S.A. **102**, 379–384 (2005). 15626758 10.1073/pnas.0406765102 PMC544290

[49] U. Truyen , Evolution of the feline-subgroup parvoviruses and the control of canine host range *in vivo*. J. Virol. **69**, 4702–4710 (1995). 7609035 10.1128/jvi.69.8.4702-4710.1995 PMC189276

[50] D. B. Tresnan, L. Southard, W. Weichert, J. Y. Sgro, C. R. Parrish, Analysis of the cell and erythrocyte binding activities of the dimple and canyon regions of the canine parvovirus capsid. Virology **211**, 123–132 (1995). 7645206 10.1006/viro.1995.1385

[51] A. B. Allison , Single mutations in the VP2 300 loop region of the three-fold spike of the carnivore parvovirus capsid can determine host range. J. Virol. **90**, 753–767 (2016). 26512077 10.1128/JVI.02636-15 PMC4702700

[52] J. E. Sykes, C. R. Parrish, “30–Feline panleukopenia virus infection and other feline viral enteritides” in Greene’s Infectious Diseases of the Dog and Cat (Fifth Edition), J. E. Sykes, Ed. (W.B. Saunders, Philadelphia, 2021), pp. 352–359.

[53] S. R. Hartmann , Cryo EM structures map a post vaccination polyclonal antibody response to canine parvovirus. Commun. Biol. **6**, 955 (2023). 37726539 10.1038/s42003-023-05319-7 PMC10509169

[54] O. F. Adu , Structures and functions of the limited natural polyclonal antibody response to parvovirus infection. Proc. Natl. Acad. Sci. U.S.A. **122**, e2423460122 (2025). 39951487 10.1073/pnas.2423460122 PMC11873831

[55] H. M. Callaway , Complex and dynamic interactions between parvovirus capsids, transferrin receptors, and antibodies control cell infection and host range. J. Virol. **92**, e00460-18 (2018). 29695427 10.1128/JVI.00460-18 PMC6002733

[56] L. Govindasamy, K. Hueffer, C. R. Parrish, M. Agbandje-McKenna, Structures of host range-controlling regions of the capsids of canine and feline parvoviruses and mutants. J. Virol. **77**, 12211–12221 (2003). 14581558 10.1128/JVI.77.22.12211-12221.2003 PMC254256

[57] Y. J. Kim , Genetic characterization of feline parvovirus isolate Fe-P2 in Korean cat and serological evidence on its infection in wild leopard cat and Asian badger. Front. Vet. Sci. **8**, 650866 (2021). 34026890 10.3389/fvets.2021.650866 PMC8138573

[58] D.-J. An , Phylogenetic analysis of feline panleukopenia virus (FPLV) strains in Korean cats. Res. Vet. Sci. **90**, 163–167 (2011). 20627272 10.1016/j.rvsc.2010.05.010

[59] E. P. Lane , Feline panleukopaenia virus in captive non-domestic felids in South Africa. Onderstepoort J. Vet. Res. **83**, a1099 (2016). 27380652 10.4102/ojvr.v83i1.1099 PMC6238724

[60] E. Kwan , Analysis of canine parvoviruses circulating in Australia reveals predominance of variant 2b and identifies feline parvovirus-like mutations in the capsid proteins. Transbound. Emerg. Dis. **68**, 656–666 (2021). 32657506 10.1111/tbed.13727

[61] D. O. Oluwayelu , Genetic characterization of parvoviruses identified in stray cats in Nigeria. Acta Trop. **250**, 107108 (2024). 38145830 10.1016/j.actatropica.2023.107108

[62] X. Chen , Circulation of heterogeneous Carnivore protoparvovirus 1 in diarrheal cats and prevalence of an A91S feline panleukopenia virus variant in China. Transbound. Emerg. Dis. **69**, e2913–e2925 (2022). 35737580 10.1111/tbed.14641

[63] R. Wang , Genetic analysis of feline parvovirus reveals predominance of feline parvovirus-G1 group among cats in China. J. Vet. Med. Sci. **86**, 1032–1039 (2024). 39010245 10.1292/jvms.24-0138 PMC11422690

[64] I. E. H. Voorhees , Limited intrahost diversity and background evolution accompany 40 years of canine parvovirus host adaptation and spread. J. Virol. **94**, e01162-19 (2019). 31619551 10.1128/JVI.01162-19 PMC6912114

[65] S. Guindon , New algorithms and methods to estimate maximum-likelihood phylogenies: Assessing the performance of PhyML 3.0. Syst. Biol. **59**, 307–321 (2010). 20525638 10.1093/sysbio/syq010

[66] J. Trifinopoulos, L.-T. Nguyen, A. von Haeseler, B. Q. Minh, W-IQ-TREE: A fast online phylogenetic tool for maximum likelihood analysis. Nucleic Acids Res. **44**, W232–W235 (2016). 27084950 10.1093/nar/gkw256 PMC4987875

[67] A. Rambaut, T. T. Lam, L. Max. Carvalho, O. G. Pybus, Exploring the temporal structure of heterochronous sequences using TempEst (formerly Path-O-Gen). Virus Evol. **2**, vew007 (2016). 27774300 10.1093/ve/vew007 PMC4989882

[68] M. A. Suchard , Bayesian phylogenetic and phylodynamic data integration using BEAST 1.10. Virus Evol. **4**, vey016 (2018). 29942656 10.1093/ve/vey016 PMC6007674

[69] A. Rambaut, A. J. Drummond, D. Xie, G. Baele, M. A. Suchard, Posterior summarization in Bayesian phylogenetics using Tracer 1.7. Syst. Biol. **67**, 901–904 (2018). 29718447 10.1093/sysbio/syy032 PMC6101584

[70] S. Weaver , Datamonkey 2.0: A modern web application for characterizing selective and other evolutionary processes. Mol. Biol. Evol. **35**, 773–777 (2018). 29301006 10.1093/molbev/msx335 PMC5850112

[71] J. Abramson , Accurate structure prediction of biomolecular interactions with AlphaFold 3. Nature **630**, 493–500 (2024). 38718835 10.1038/s41586-024-07487-w PMC11168924

[72] S.-J. Park , CHARMM-GUI Glycan Modeler for modeling and simulation of carbohydrates and glycoconjugates. Glycobiology **29**, 320–331 (2019). 30689864 10.1093/glycob/cwz003 PMC6422236

[73] E. F. Pettersen , UCSF ChimeraX: Structure visualization for researchers, educators, and developers. Protein Sci. **30**, 70–82 (2021). 32881101 10.1002/pro.3943 PMC7737788

[74] D. J. Goetschius , High-resolution asymmetric structure of a Fab-virus complex reveals overlap with the receptor binding site. Proc. Natl. Acad. Sci. U.S.A. **118**, e2025452118 (2021). 34074770 10.1073/pnas.2025452118 PMC8201801

[75] L. J. Organtini , Near-atomic resolution structure of a highly neutralizing Fab bound to Canine Parvovirus. J. Virol. **90**, 9733–9742 (2016). 27535057 10.1128/JVI.01112-16 PMC5068525

[76] D. Wang, W. Yuan, I. Davis, C. R. Parrish, Nonstructural protein-2 and the replication of canine parvovirus. Virology **240**, 273–281 (1998). 9454701 10.1006/viro.1997.8946

[77] Z. Zádori, J. Szelei, P. Tijssen, SAT: A late NS protein of porcine parvovirus. J. Virol. **79**, 13129–13138 (2005). 16189014 10.1128/JVI.79.20.13129-13138.2005 PMC1235825

[78] P. J. Young, K. T. Jensen, L. R. Burger, D. J. Pintel, C. L. Lorson, Minute virus of mice small nonstructural protein NS2 interacts and colocalizes with the Smn protein. J. Virol. **76**, 6364–6369 (2002). 12021369 10.1128/JVI.76.12.6364-6369.2002 PMC136193

[79] C. L. Miller, D. J. Pintel, Interaction between parvovirus NS2 protein and nuclear export factor Crm1 is important for viral egress from the nucleus of murine cells. J. Virol. **76**, 3257–3266 (2002). 11884550 10.1128/JVI.76.7.3257-3266.2002 PMC136031

[80] S. K. Tewary , Structures of minute virus of mice replication initiator protein N-terminal domain: Insights into DNA nicking and origin binding. Virology **476**, 61–71 (2015). 25528417 10.1016/j.virol.2014.11.022 PMC4699654

[81] J. P. Nüesch, S. F. Cotmore, P. Tattersall, Sequence motifs in the replicator protein of parvovirus MVM essential for nicking and covalent attachment to the viral origin: Identification of the linking tyrosine. Virology **209**, 122–135 (1995). 7747462 10.1006/viro.1995.1236

[82] N. Inthong , Feline panleukopenia virus as the cause of diarrhea in a banded linsang (*Prionodon linsang*) in Thailand. J. Vet. Med. Sci. **81**, 1763–1768 (2019). 31548471 10.1292/jvms.19-0238 PMC6943334

[83] S. Yang , Isolation and characterization of feline panleukopenia virus from a diarrheic monkey. Vet. Microbiol. **143**, 155–159 (2010). 20044220 10.1016/j.vetmic.2009.11.023 PMC7117194

[84] S. Yi , Feline panleukopenia virus with G299E substitution in the VP2 protein first identified from a captive Giant Panda in China. Front. Cell. Infect. Microbiol. **11**, 820144 (2021). 35198456 10.3389/fcimb.2021.820144 PMC8859993

[85] Y. Yang , Identification of a Feline Panleukopenia Virus from Captive Giant Pandas (Ailuropoda melanoleuca) and Its Phylogenetic Analysis. Transbound. Emerg. Dis. **2023**, 7721487 (2023). 40303714 10.1155/2023/7721487 PMC12016729

[86] P. Simmonds, P. Aiewsakun, A. Katzourakis, Prisoners of war–Host adaptation and its constraints on virus evolution. Nat. Rev. Microbiol. **17**, 321–328 (2019). 30518814 10.1038/s41579-018-0120-2 PMC7097816

[87] D. S. McBride , Accelerated evolution of SARS-CoV-2 in free-ranging white-tailed deer. Nat. Commun. **14**, 5105 (2023). 37640694 10.1038/s41467-023-40706-y PMC10462754

[88] M. Worobey, G.-Z. Han, A. Rambaut, A synchronized global sweep of the internal genes of modern avian influenza virus. Nature **508**, 254–257 (2014). 24531761 10.1038/nature13016 PMC4098125

[89] A. B. Allison , Role of multiple hosts in the cross-species transmission and emergence of a pandemic parvovirus. J. Virol. **86**, 865–872 (2012). 22072763 10.1128/JVI.06187-11 PMC3255849

[90] A. A. Guzmán-Solís , Ancient viral genomes reveal introduction of human pathogenic viruses into Mexico during the transatlantic slave trade. Elife **10**, e68612 (2021). 34350829 10.7554/eLife.68612 PMC8423449

[91] B. Mühlemann , Ancient human parvovirus B19 in Eurasia reveals its long-term association with humans. Proc. Natl. Acad. Sci. U.S.A. **115**, 7557–7562 (2018). 29967156 10.1073/pnas.1804921115 PMC6055166

[92] A. B. Allison , Frequent cross-species transmission of parvoviruses among diverse carnivore hosts. J. Virol. **87**, 2342–2347 (2013). 23221559 10.1128/JVI.02428-12 PMC3571474

[93] S. N. Emmanuel, M. Mietzsch, Y. S. Tseng, J. K. Smith, M. Agbandje-McKenna, Parvovirus capsid-antibody complex structures reveal conservation of antigenic epitopes across the family. Viral Immunol. **34**, 3–17 (2021). 32315582 10.1089/vim.2020.0022 PMC8020512

[94] A. L. Llamas-Saiz , Structural analysis of a mutation in canine parvovirus which controls antigenicity and host range. Virology **225**, 65–71 (1996). 8918534 10.1006/viro.1996.0575

[95] R. A. López-Astacio , Viral capsid, antibody, and receptor interactions: Experimental analysis of the antibody escape evolution of Canine Parvovirus. J. Virol. **97**, e0009023 (2023). 37199627 10.1128/jvi.00090-23 PMC10308881

[96] C. R. Parrish, Pathogenesis of feline panleukopenia virus and canine parvovirus. Baillieres Clin. Haematol. **8**, 57–71 (1995). 7663051 10.1016/S0950-3536(05)80232-X PMC7134857

[97] P. C. Meunier, B. J. Cooper, M. J. Appel, D. O. Slauson, Pathogenesis of canine parvovirus enteritis: The importance of viremia. Vet. Pathol. **22**, 60–71 (1985). 2983478 10.1177/030098588502200110

[98] C. E. Z. Chan , Residual humoral immunity sustained over decades in a cohort of vaccinia-vaccinated individuals. J. Infect. Dis. **227**, 1002–1006 (2023). 36200239 10.1093/infdis/jiac409

[99] M. Á. Muñoz-Alía, R. A. Nace, L. Zhang, S. J. Russell, Serotypic evolution of measles virus is constrained by multiple co-dominant B cell epitopes on its surface glycoproteins. Cell Rep. Med. **2**, 100225 (2021). 33948566 10.1016/j.xcrm.2021.100225 PMC8080110

[100] R. L. de Swart, W. P. Duprex, A. D. M. E. Osterhaus, Rinderpest eradication: Lessons for measles eradication? Curr. Opin. Virol. **2**, 330–334 (2012). 22709518 10.1016/j.coviro.2012.02.010

[101] E. Waller, Infectious gastroenteritis in raccoons (*Procyon lotor*). J. Am. Vet. Med. Assoc. **96**, 266–268 (1940).

[102] L. A. Shackelton, E. C. Holmes, Phylogenetic evidence for the rapid evolution of human B19 erythrovirus. J. Virol. **80**, 3666–3669 (2006). 16537636 10.1128/JVI.80.7.3666-3669.2006 PMC1440363

[103] J. D. Tratschin, G. K. McMaster, G. Kronauer, G. Siegl, Canine parvovirus: Relationship to wild-type and vaccine strains of feline panleukopenia virus and mink enteritis virus. J. Gen. Virol. **61**, 33–41 (1982). 6181186 10.1099/0022-1317-61-1-33

[104] U. Truyen, K. Geissler, C. R. Parrish, W. Hermanns, G. Siegl, No evidence for a role of modified live virus vaccines in the emergence of canine parvovirus. J. Gen. Virol. **79**, 1153–1158 (1998). 9603330 10.1099/0022-1317-79-5-1153

[105] A. B. Allison , Host-specific parvovirus evolution in nature is recapitulated by in vitro adaptation to different carnivore species. PLoS Pathog. **10**, e1004475 (2014). 25375184 10.1371/journal.ppat.1004475 PMC4223063

[106] S. F. Cotmore, P. Tattersall, “Chapter 29. Parvovirus” in DNA Replication and Human Disease, M. L. DePamphilis, Ed. (Cold Spring Harbor Laboratory Press, New York, 2006), pp. 593–608.

[107] R. A. López-Astacio , An analysis of feline parvovirus (FPV) evolution; in comparison to the emergent canine parvovirus (CPV) and an assessment of antigenic variation from vaccine strains. NCBI. https://www.ncbi.nlm.nih.gov/bioproject/PRJNA1288508. Deposited 8 July 2025.

