# Supplementary material for: Distinct evolutionary patterns of endemic and emerging parvoviruses and the origin of a new pandemic virus
Source: Proc Natl Acad Sci U S A. 2026 Apr 14;123(16):e2515274123. doi: 10.1073/pnas.2515274123 (PMC13099694; doi:10.1073/pnas.2515274123)
Supplement: Supplementary file 1 — Appendix 01 (PDF) [file pnas.2515274123.sapp.pdf]

Supporting Information for
Distinct evolutionary patterns of endemic and emerging parvoviruses, and the origin of a new pandemic virus.

Robert A. López-Astacio, Brian R. Wasik, Hyunwook Lee, Ian E. H. Voorhees, Wendy S. Weichert, Oluwafemi F. Adu, Laura B. Goodman, Susan L. Hafenstein, Uwe Truyen, Colin R. Parrish

Colin R. Parrish
Email: crp3@cornell.edu

This PDF file includes:

Tables S1 to S3

Tables

Table S1. Metadata of sequences used in this study, including sample ID, virus species, clade designation, source of sample, host of origin, collection year, geographic origin, and GenBank accession #s.

Sample ID	Virus	Clade	Source	Host	Collection Year	Country ISO	GenBank Accession
PureVax	FPV		Live-attenuated vaccine				PV936305
Solo-Jec Feline 3	FPV		Live-attenuated vaccine				PV936307
Virbagen Felis RCP	FPV		Live-attenuated vaccine				PV936309
Versifel CVR-T	FPV		Live-attenuated vaccine				PV936308
PureVax RCP	FPV		Live-attenuated vaccine				PV936306
Nobivac RCP	FPV		Live-attenuated vaccine				PV936304
FPV-1	FPV		Feline kidney cell culture		1964	US	PV936289
FPV-2	FPV		Feline cell culture		1965	US	PV936296
FPV-LV	FPV		Feline cell culture		1970	DE	PV936302
FPV-193/70	FPV		Feline embryo cell culture		1973	AU	X55115
CU-4	FPV	A	Feline kidney cell culture		1982	US	M38246
FPV-10	FPV	A	Feline kidney cell culture		1991	US	PV936290
Gigucheon	FPV	B	Feline kidney cell culture		2017	KR	MN400978
TN/FPV/2018	FPV	B	Feline cell culture		2018	IN	MH559110
FPV-1/97	FPV		Vaccine-derived, host	Domestic Cat	1997	ZA	PV936295
FPV-48/20	FPV		Vaccine-derived, host	Domestic Cat	2007	DE	PV936301
FPV-BJ05	FPV		Vaccine-derived, host	Domestic Cat	2014	CN	MH165482

FPV-BJ04	FPV		Vaccine-derived, host	Domestic Cat	2015	CN	MH165481
FPV-190/16	FPV		Vaccine-derived, host	Domestic Cat	2016	DE	PV936294
MAHG-3/BC_2017	FPV		Vaccine-derived, host	American Pine Marten	2017	CA	MN862745
19R124C/TH/2019	FPV		Vaccine-derived, host	Domestic Cat	2019	TH	MN127781
FPV-4	FPV		Host	Domestic Cat	1964	US	EU659112
FPV-3	FPV	A	Host	Domestic Cat	1967	US	EU659111
MEV5	FPV		Host	American Mink	1970	DE	PV936303
FAV3	FPV	A	Host	Domestic Cat	1970	DE	PV936288
BFPV	FPV		Host	Blue Arctic Fox	1983	FI	MN451652
HH-1/86	FPV	B	Host	Jaguar	1986	CN	KX900570
FPV-8a	FPV	A	Host	Mountain Lion	1989	US	EU659113
FPV-8b	FPV	A	Host	Mountain Lion	1989	US	EU659114
FPV-G	FPV	B	Host	Tiger	1999	CN	MG764510
FPV-kai	FPV	A	Host	Domestic Cat	2006	US	EU659115
FPV-270/06	FPV	B	Host	Domestic Cat	2006	DE	PV936297
FPV-2/07	FPV	B	Host	Domestic Cat	2007	DE	PV936298
XJ-1	FPV	B	Host	Leopard	2007	CN	EF988660
FPV-331/08	FPV		Host	Leopard	2008	DE	PV936299
RC9/BC_2010	FPV	A	Host	Raccoon	2010	CA	MF069446
FPV-125/11	FPV	B	Host	Domestic Cat	2011	DE	PV936291
FPV-126/11	FPV	B	Host	Domestic Cat	2011	DE	PV936292
MG132167A	FPV	B	Host	Domestic Cat	2013	BE	KP769859
FPV-183/14	FPV		Host	Domestic Cat	2014	DE	PV936293
HRB-CS1	FPV	B	Host	Domestic Cat	2014	CN	KP280068
RC6/BC_2015	FPV	A	Host	Raccoon	2015	CA	MF069445

FPV_IZSSI_3201_1_15	FPV		Host	Domestic Cat	2015	IT	KX434461
FPV-L	FPV	B	Host	Lion	2015	CN	MG764511
MAVI-36/BC_2016	FPV	A	Host	American Pine Marten	2016	CA	MN862744
RC18/BC_2016	FPV	A	Host	Raccoon	2016	CA	MF069447
HN-ZZ1	FPV	B	Host	Tiger	2016	CN	KX685354
MIVI-21/BC_2017	FPV	A	Host	American Mink	2017	CA	MN862746
MIVI-34/BC_2018	FPV	A	Host	American Mink	2018	CA	MN862743
GPFE267PAR01-12	FPV	B	Host	Giant Panda	2018	CN	MZ357122
OTVI-16/BC_2019	FPV	A	Host	North American River Otter	2019	CA	MN862748
OTVI-3/BC_2019	FPV	A	Host	North American River Otter	2019	CA	MN862749
MIVI-72/BC_2019	FPV	A	Host	American Mink	2019	CA	MN862747
MHS2019	FPV	A	Host	Tiger	2019	CN	MN908257
19R81C/TH/2019	FPV	B	Host	Domestic Cat	2019	TH	MN127780
CF094PAR01-08	FPV	A	Host	Domestic Cat	2020	CN	MZ357120
FPV-SH2003	FPV	A	Host	Domestic Cat	2020	CN	MW811187
FPV-SH2001	FPV	A	Host	Domestic Cat	2020	CN	MW650831
RPFE013PAR01-05	FPV	B	Host	Red Panda	2020	CN	MZ357119
GPFE016PAR01-05	FPV	B	Host	Giant Panda	2020	CN	MW331496
FPV-38/21	FPV	A	Host	Domestic Cat	2021	DE	PV936300
CPV12	CPV		Host	Domestic Dog	1978	US	MN451655
CPV6	CPV		Host	Domestic Dog	1979	US	MN451653
CPV9	CPV		Host	Domestic Dog	1979	US	MN451654
CPV5	CPV		Host	Domestic Dog	1980	US	EU659116
CPV63	CPV		Host	Domestic Dog	1980	US	MN451668

Table S2. FPV isolates directly sequenced in this study: ID, SRA accession #s, NCBI BioSample accession #s, and GenBank accession #s.

Isolate ID	SRA Experiment	SRA Run	BioSample	GenBank
FAV3	SRX29591405	SRR34430127	SAMN49851095	PV936288
FPV-1	SRX29591417	SRR34430115	SAMN49851087	PV936289
FPV-10	SRX29591420	SRR34430112	SAMN49851090	PV936290
FPV-125/11	SRX29591409	SRR34430123	SAMN49851099	PV936291
FPV-126/11	SRX29591410	SRR34430122	SAMN49851100	PV936292
FPV-183/14	SRX29591412	SRR34430120	SAMN49851101	PV936293
FPV-190/16	SRX29591403	SRR34430129	SAMN49851093	PV936294
FPV-1/97	SRX29591401	SRR34430131	SAMN49851091	PV936295
FPV-2	SRX29591418	SRR34430114	SAMN49851088	PV936296
FPV-270/06	SRX29591406	SRR34430126	SAMN49851096	PV936297
FPV-2/07	SRX29591407	SRR34430125	SAMN49851097	PV936298
FPV-331/08	SRX29591408	SRR34430124	SAMN49851098	PV936299
FPV-38/21	SRX29591413	SRR34430119	SAMN49851102	PV936300
FPV-48/20	SRX29591402	SRR34430130	SAMN49851092	PV936301
FPV-LV	SRX29591419	SRR34430113	SAMN49851089	PV936302
MEV5	SRX29591404	SRR34430128	SAMN49851094	PV936303
NobivacRCP	SRX29591416	SRR34430116	SAMN49851086	PV936304
PureVax	SRX29591399	SRR34430133	SAMN49851081	PV936305
PureVaxRCP	SRX29591415	SRR34430117	SAMN49851085	PV936306
Solo-JecFeline3	SRX29591400	SRR34430132	SAMN49851082	PV936307
VersifelCVR-T	SRX29591414	SRR34430118	SAMN49851084	PV936308
VirbagenFelisRCP	SRX29591411	SRR34430121	SAMN49851083	PV936309

Table S3. Widespread variants among FPV wildtype natural infection samples (*n*=40).

Nucleotide	s/ns	ORF	Amino Acid	% samples
G54A	s	NS1	L18	25
A67G	ns	NS1	N23D	42.5
T486G	s	NS1	L162	12.5
T489C	s	NS1	C163	10
G493A	ns	NS1	V165I	15
G555A	s	NS1	V185	10
T741A	ns	NS1	H247Q	45
G849T	s	NS1	V283	27.5
C906T	s	NS1	D302	25
G1251A	s	NS1	V417	15
T1254G	s	NS1	G418	32.5
T1266C	s	NS1	C422	12.5
A1327G	ns	NS1	I433V	47.5
T1431A	s	NS1	I477	12.5
C1633G	ns	NS1	Q545E	57.5
T1653C	s	NS1	Y551	35
A1680G	s	NS1	E560	37.5
G1763A	ns	NS1	S588N	15
C1785A	ns	NS1/NS2	H595Q/R105S	45
G1786C	ns	NS1/NS2	V596L/R105T	10
A1926G	ns	NS2	M152V	40
T1959C	ns	NS2	F163L	57.5
G2080A	s	intron		37.5
T2083A	s	intron		10
C2110T	s	intron		12.5
T2114C	s	intron		10
T2262C	s	VP1	Y59	47.5
C2265T	s	VP1	F60	52.5
A2355G	s	VP1	K90	47.5
G2658A	ns	SAT	R46K	12.5
A2685G	ns	SAT	D55G	45
G2785T	ns	VP2	A91S	12.5
A3153G	s	VP2	Q213	12.5
G3264A	s	VP2	V250	10
G3369A	s	VP2	L285	12.5
C3385T	s	VP2	L291	52.5
C3420T	s	VP2	N302	12.5
A3468G	s	VP2	Q318	10
T3504A	s	VP2	I330	12.5
G3555A	s	VP2	A347	55
A3663G	s	VP2	Q383	10
T3699C	s	VP2	P385	32.5
G4035A	s	VP2	T507	65
A4080G	s	VP2	V522	35
T4086C	s	VP2	Y524	62.5
